# Co-Operation or Chaos?

**Published:** 1921-01-08

**Authors:** 


### The Hospital

The Workers' Newspaper of Administrative Medicine and Institutional Life, Administration, National Insurance and Health.

No. 1805, Vol. LXIX. SATURDAY, JANUARY 8, 1921. PRICE TWOPENCE.

### CO-OPERATION OR CHAOS?

Following upon the letter (which we published on December 25) from the Secretary of the Federation of Medical and Allied Societies, it may be well closely to consider the position which his concluding paragraph indicates. To make the particular issue clearer, let us quote the essential words. With regard to this Federation, "among the forty-five organisations co-operating at the present time . . . as all the allied professions are fully represented it requires only the co-operation of the British Medical Association and the Medico-Political Union to make adequate representation of medicine possible. Is not this a goal worth some personal sacrifice?"

In answer to this question we believe that our readers, medical and lay, will most heartily agree that anything which will allow of the expression of really representative professional opinion is worth more, indeed, than mere personal sacrifice. It is worth whole-hearted and general support for any organisation which can genuinely claim the power to bring about this unity.

Also we believe that those who followed Lieut.-Col. N. Howard Mummery's valuable address delivered before the Harrogate Medical Society on November 25, 1920, and recently published in full in the *Lancet*, cannot have failed to realise the possibilities which lie before the Federation if helped and not hindered in its work. It appears to us to be a question of providing a great public benefit, quite apart from the purely professional aspects of the case.

Dr. Addison has himself put the situation perfectly clearly to the doctors. They must set up some machinery whereby they can be consulted. The multiplicity of associations and professional societies, as well as its resulting disadvantages, are obvious to the most disinterested observer, and, as these units are nowhere linked by a common channel of official representation, the impossibility is equally apparent of making genuine use of Dr. Addison's invitation and his definite agreement as to the reasonableness of consultation during the formative stages of legislation. But even were the doctors, as such, so organised that criticism of their societies were unjustifiable, still there remains a host of workers in other professions, but quite inseparable from correct and modern health administration, and it is an outstanding fact that no purely medical association can bring together all these diverse units.

Therefore, without attempting to enter into the reasons that may or may not cause the British Medical Association and the smaller Medico-Political Union to withhold their support from the one organisation which, as it appears to us, has, at the moment, this power to initiate the wider type of professional co-operation, we will simply recount certain facts in connection with the avowed policy of the Federation, and leave our readers to judge for themselves.

In such an organisation it is not intended that any one body can be dominant. Federation means federation on equal terms, except that representation in proportion to numbers or importance is possible. The office of the Federation is run apart from any of the constituent bodies, and, since the policy of the constituent bodies automatically makes that of the Federation, the latter has, strictly speaking, no policy of its own, and therefore should have no direct opponents. In event of disagreement among its constituent bodies its duty is (*a*) to attempt reconciliation of their views, or (*b*) to announce divergence of views if they are found to be irreconcilable. A Federation as such is not called upon to *lead* deputations or to argue with authorities, central or local. When a deputation is necessary it would be led by the chief body primarily concerned, the others lending their support. In event of complete divergence of opinion it is quite possible that two deputations might approach the authority (*e.g.*, the venereal diseases question), and it must be realised that a federation is impartial and exists merely for the purpose of organising, collating, and focussing opinions.

It will be admitted that the medical profession urgently needs a co-ordinative force of this type. But, again, let us remember that in dealing with national health questions the constituent bodies of a federation must not be those representing qualified medical men alone. We see it stated that the British Medical Association has under consideration a plan for so altering its constitution that it may become a federation of bodies as well as an association of individuals. In existing circumstances we fail to see how such a move on the part of the leading medical association can meet all the complex requirements of the times, when health questions require for their solution so many types of expert knowledge and experience. To us it appears that the nation has at hand the mechanism for a possible collective representation of the professions concerned. Would it not be better for the existing societies to concentrate, as heretofore, on their own special spheres and to support and use when necessary the new means of co-operation opened to them? We think so. But it is a question for their members to decide, and, in view of the short time available before the question of new health legislation is again to the front, we trust they will lose no opportunity of expressing their opinions.

